# Case report: a novel deep intronic splice-altering variant in *DMD* as a cause of Becker muscular dystrophy

**DOI:** 10.3389/fgene.2023.1226766

**Published:** 2023-09-19

**Authors:** Shala Ghaderi Berntsson, Hans Matsson, Anna Kristoffersson, Valter Niemelä, Hermine A. van Duyvenvoorde, Cindy Richel-van Assenbergh, Heleen M. van der Klift, Olivera Casar-Borota, Carina Frykholm, Anne-Marie Landtblom

**Affiliations:** ^1^ Department of Medical Sciences, Neurology, Uppsala University, Uppsala, Sweden; ^2^ Department of Immunology, Genetics and Pathology, Uppsala University, Uppsala, Sweden; ^3^ Clinical Genetics, Rudbeck Laboratory, Uppsala University Hospital, Uppsala, Sweden; ^4^ Department of Clinical Genetics, Leiden University Medical Center, Leiden, Netherlands; ^5^ Department of Clinical Pathology, Uppsala University Hospital, Uppsala, Sweden; ^6^ Department of Clinical Department of Biomedical and Clinical Sciences, Faculty of Medicine and Health, Linköping University, Linköping, Sweden

**Keywords:** Becker muscular dystrophy, genetics, MLPA, mRNA, RNA sequencing, intronic variant

## Abstract

We present the case of a male patient who was ultimately diagnosed with Becker muscular dystrophy (BMD; MIM# 300376) after the onset of muscle weakness in his teens progressively led to significant walking difficulties in his twenties. A genetic diagnosis was pursued but initial investigation revealed no aberrations in the dystrophin gene (*DMD*), although immunohistochemistry and Western blot analysis suggested the diagnosis of dystrophinopathy. Eventually, after more than 10 years, an RNA analysis captured abnormal splicing where 154 nucleotides from intron 43 were inserted between exon 43 and 44 resulting in a frameshift and a premature stop codon. Normal splicing of the *DMD* gene was also observed. Additionally, a novel variant c.6291–13537A>G in *DMD* was confirmed in the genomic DNA of the patient. The predicted function of the variant aligns with the mRNA results. To conclude, we here demonstrate that mRNA analysis can guide the diagnosis of non-coding genetic variants in *DMD*.

## Introduction

Becker muscular dystrophy (BMD) and Duchenne muscular dystrophy (DMD) are X-linked recessive disorders that are caused by pathogenic variants in the *DMD* gene coding for the dystrophin protein. The dystrophin protein is part of a protein complex that connects the contractile myofilaments to the extracellular matrix and stabilizes the skeletal muscle cells during contraction, protecting them from damage. A deficiency of dystrophin leads to muscle damage, inflammation, and progressive replacement of muscle tissue with connective tissue and fat over time (Ferlini et al., 2013; Aartsma-Rus et al., 2016). Pathogenic variants in the gene can also cause X-linked dilated cardiomyopathy affecting the heart muscle (Aartsma-Rus et al., 2016; Fratter et al., 2020). Rare presentations, such as isolated elevated creatine kinase levels and quadriceps myopathy, have also been associated with *DMD* gene variants (Fratter et al., 2020). The clinical symptoms of BMD begin in teens or early adulthood and appear as progressive muscle weakness. Patients have difficulty climbing stairs and must use their arms when trying to stand up due to weak thigh muscles (Gowers’s sign) (Birnkrant et al., 2018). An elevated serum creatine kinase supports the diagnosis (Fratter et al., 2020). Compared to DMD, BMD exhibits a later onset of symptoms and generally slower disease progression due to partial functionality of the dystrophin protein (Aartsma-Rus et al., 2016). The prevalence of BMD in Sweden is 4/100,000 males with an annual incidence of four boys or men^1^. The global average prevalence of BMD is 1.6/100,000 according to a meta-analysis (Salari et al., 2022).

The *DMD* gene, located on the short arm of the X chromosome, is the largest in the human genome. It consists of over 2.2 megabases (Mb) including 79 protein-coding exons. More than 99% of the gene consists of non-coding intronic regions (Keegan, 2020). The mutation rate in *DMD* is relatively high, with approximately two-third of cases inherited from the mothers, while one-third of patients have *de novo* pathogenic variants (Bladen et al., 2015; Aartsma-Rus et al., 2016). DMD and BMD affect mainly the male offspring who are hemizygous for the pathogenic gene variant (Grimm et al., 2012). Intronic variants may be underrepresented because they are more difficult to detect (Aartsma-Rus et al., 2016). In > 90% of cases, variants that disrupt the reading frame, lead to DMD, while variants that preserve the reading frame, lead to BMD (Ferlini et al., 2013; Aartsma-Rus et al., 2016).

Learning- and behavioral problems have been reported for patients with or without intellectual disabilities. Reading difficulties, impaired verbal working memory, and processing of information have been described. Comorbidity with attention deficit hyperactivity disorder (ADHD), obsessive-compulsive disorder, anxiety, autism, and epilepsy have been reported with varying prevalence. The prevalence of learning disabilities, attention- and behavioral problems, and autism in patients with BMD was higher compared to the general population. However, the mean IQ (95,6) was similar (Young et al., 2008).

This case highlights the challenges encountered in reaching a definitive genetic diagnosis of Becker muscular dystrophy. The crucial interplay between disciplines and alternative methodologies, and the addition of mRNA diagnostics ultimately helped resolve the diagnostic dilemma.

### Patient information

A young male patient born in 1997, with no known family history of muscle disease, was recently found to have Becker muscular dystrophy, caused by a rare variant in the *DMD* gene. The mother’s pregnancy and delivery were normal and during his early childhood, he reached typical physical milestones and showed no signs of muscle weakness. However, at age six, the school health service reported that he did not put his heels on the floor when walking and his feet were positioned slightly inward. Furthermore, he tripped easily, and his proximal thigh muscles were underdeveloped. Reading and learning disabilities were detected at the same time for which he received assistance until the last year of primary school. At the age of 12, he was referred for a pediatric neurology assessment at the university hospital. His parents had always perceived him as clumsy and weak in his legs, but over time they noticed that he had increasing difficulty getting up from a seated position. He could run short distances, climb stairs if the steps were not too high, and lift a milk carton without difficulties. However, he had difficulty lifting heavy grocery bags and keeping up with physical activities like school sports. He experienced pain if he walked too much and sometimes had muscle cramps after physical activities. He had found a way to cope with certain movements such as rising from a sitting position. After primary school, he attended regular school and later finished secondary school at a normal age.

Upon examination in 2010, the pediatric neurologist found normal upper extremity strength but decreased lower extremity strength bilaterally, a positive Gowers’s sign, and pseudo-hypertrophy in the calves. He had significantly increased lordosis in the lumbar spine, but no scoliosis. There was some tightness over the gastrocnemius. His creatine kinase level was significantly elevated, indicating muscle damage. Echocardiography showed no evidence of cardiomyopathy. Based on the clinical phenotype, BMD was suspected, and a genetic investigation was initiated (see below for details). Since the initial investigation did not confirm BMD, the diagnosis of limb-girdle muscle dystrophy of unknown type was proposed. The patient underwent annual monitoring of cardiac and respiratory functions, as well as regular assessments by physiotherapists and occupational therapists. A new diagnostic investigation was initiated, and the disease progressed slowly over time, with the patient experiencing significant difficulty in walking in his early twenties. He was able to work part-time repairing vehicles, with adapted tasks, and he had a driving license. He was still actively working on a thorough clinical follow-up in autumn 2021. A renewed cardiology investigation revealed no gross pathology, but due to the increased risk for dilated cardiomyopathy, a cardiology examination was recommended with follow-up examinations annually or biannually.

### Diagnostic assessment

**FIGURE 1:**
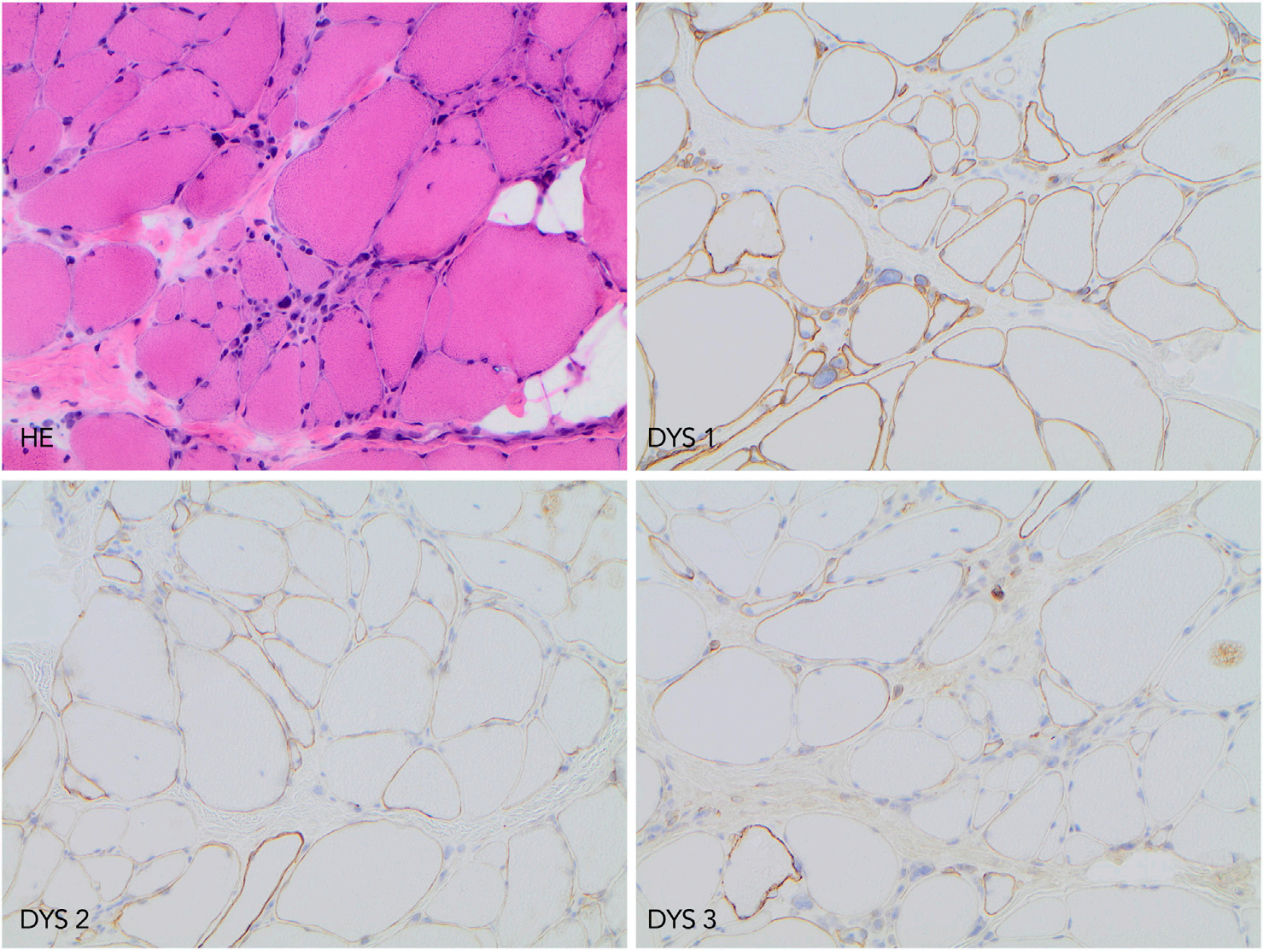
Histopathology and immunohistochemistry findings. Upper left image, hematoxylin-eosin (HE) staining demonstrating chronic myopathy features. The upper right image shows preserved immunolabeling when using antibody DYS1. In lower left and right images, reduced dystrophin protein expression was detected using antibodies DYS 2 and DYS 3. All microphotographs were taken at ×200 magnification.

Histological, immunohistochemistry, and Western blot investigations (Department of Clinical Pathology and Cytology, Sahlgrenska University Hospital, Gothenburg) were performed on a muscle biopsy taken from the patient´s thigh in 2010. The findings indicated myopathy with partial dystrophin deficiency. In 2015, re-analyses of histology and immunohistochemistry were performed at the Department of Pathology, Uppsala University Hospital. A variability of muscle fiber diameter, groups of regenerated myofibers, fibrosis, fat deposition, internalized nuclei, and scattered necrotic myofibers was noted. Immunohistochemistry showed normal levels of dystrophin using an antibody targeting the dystrophin rod domain (NCL-DYS1, Novocastra) but reduced levels using antibodies for the dystrophin C- and N-terminus (NCL-DYS2 and NCL-DYS3, Novocastra), respectively, (Figure 1). Western blot investigation using (NCL-DYS2, Novocastra) antibody reported decreased levels of dystrophin but with the same molecular weight as a control sample.

Multiplex ligation-dependent probe amplification (MLPA; SALSA P034/P035 MRC-Holland) performed at Clinical Genetics, Uppsala University Hospital detected no deletions nor duplications in the *DMD* gene. Subsequent sequencing of *DMD* (including all 79 protein-coding exons, canonical splice sites, and approximately 50 nucleotides of flanking intronic sequences) performed at the Department of Human Genetics, University of Utah, US showed no pathogenic variants. Hence, routine genetic testing could not confirm the clinical BMD diagnosis, and a diagnosis of limb-girdle muscle dystrophy of unknown type was proposed. A Muscular Dystrophy gene panel was not available in 2010, and no other specific gene was tested. A test for protein truncation was discussed but never performed, since the laboratory (Clinical Genetics, Sahlgrenska University Hospital) had discontinued the method.

In 2015, the previous analyses were repeated on the same muscle sample. Initially with immunohistochemistry at the Department of Pathology (University Hospital, Uppsala) and for an extended Western blot analysis the same muscle sample was sent abroad (Centre de Pathology Est, Hospices Civils de Lyon). The results were not conclusive and relative quantification of dystrophin protein level was not available.

**FIGURE 2:**
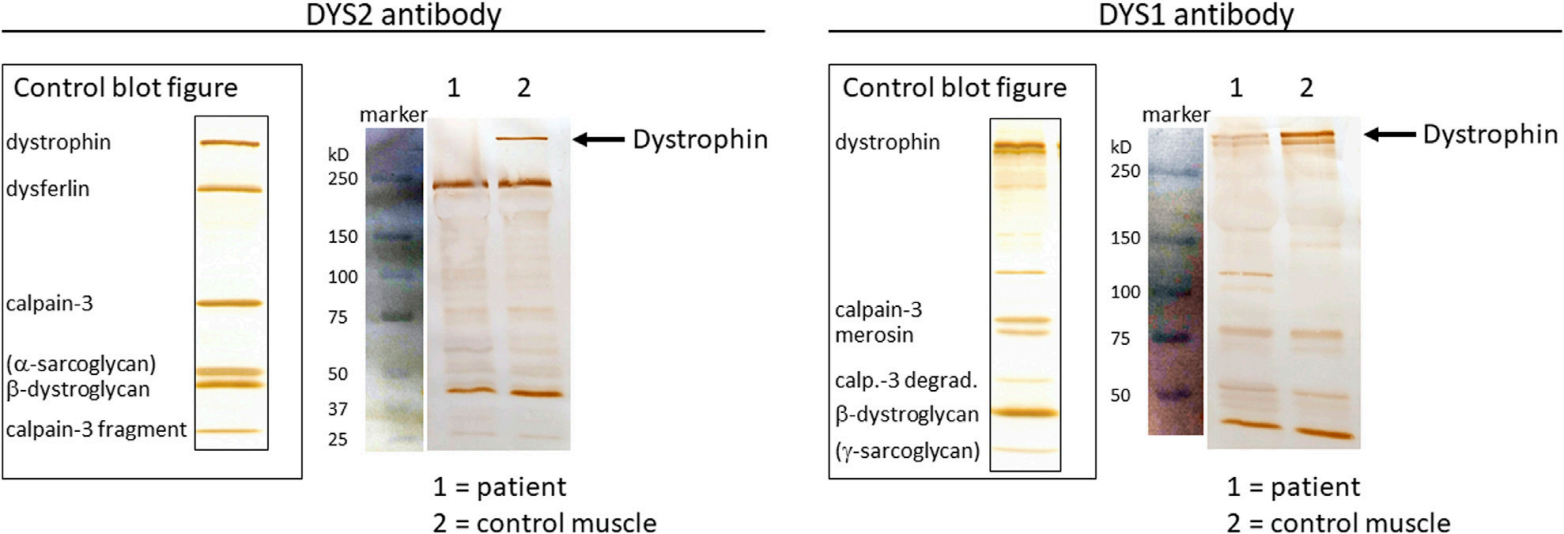
Protein analyses from muscle tissue biopsy using Western blotting. Control blot figures are presented together with separated protein extracts from patient biopsy and control muscle tissue using the DYS2 antibody (left panel) and the DYS1 antibody (right panel). Protein size markers (blue-colored bands) are shown with corresponding kilodaltons (kD) marker sizes. Full-length DMD protein (400 kD) is depicted with black arrows.

**FIGURE 3:**
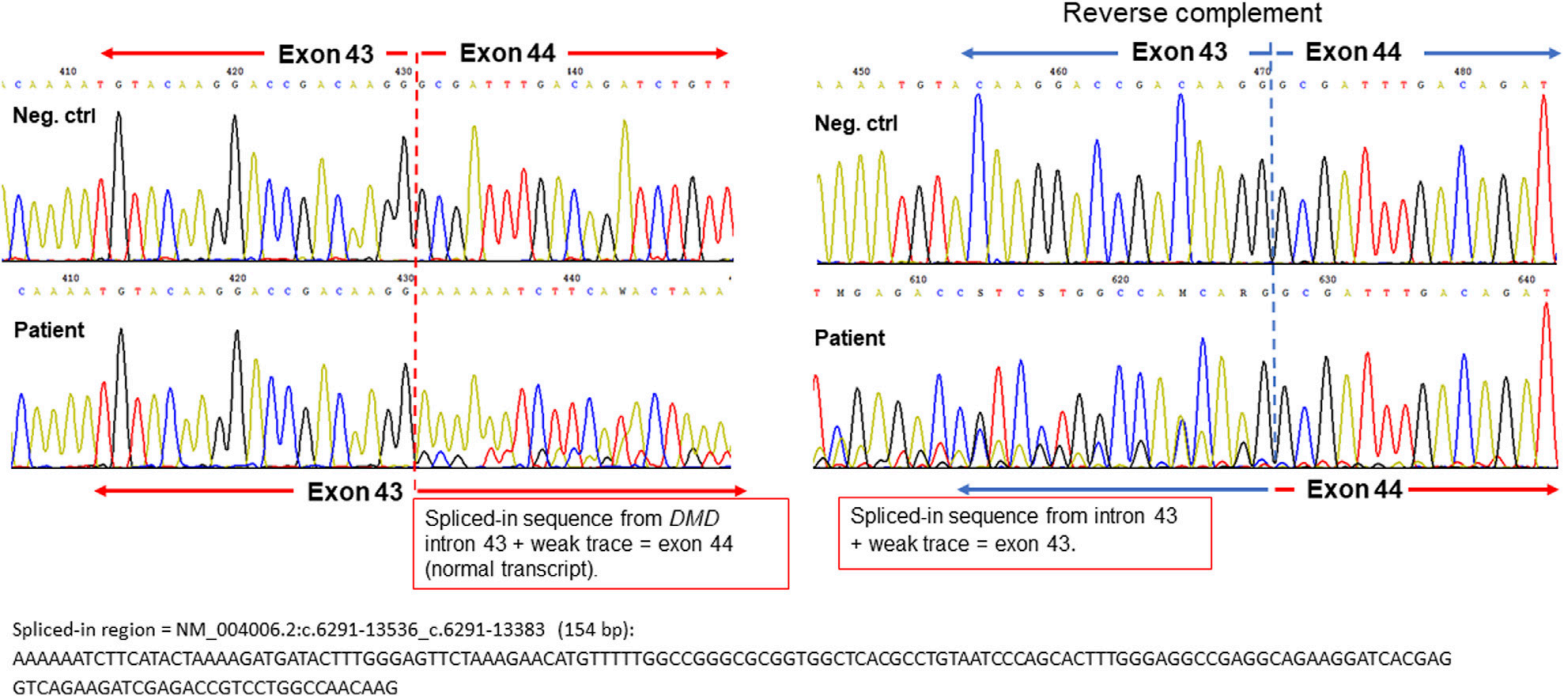
Sequencing chromatograms of the beginning of the spliced-in *DMD* intron 43 sequence (aberrant transcript) from the forward strand (left panel) and reverse complement strand (right panel). Negative controls are shown as upper sequence traces and the *DMD* allele from the patient as lower traces for comparison. The start position of the partial intron retention in *DMD* intron 43 is depicted with dashed red and blue lines, respectively. The full nucleotide sequence of the spliced-in region is shown below the chromatograms.

**FIGURE 4:**
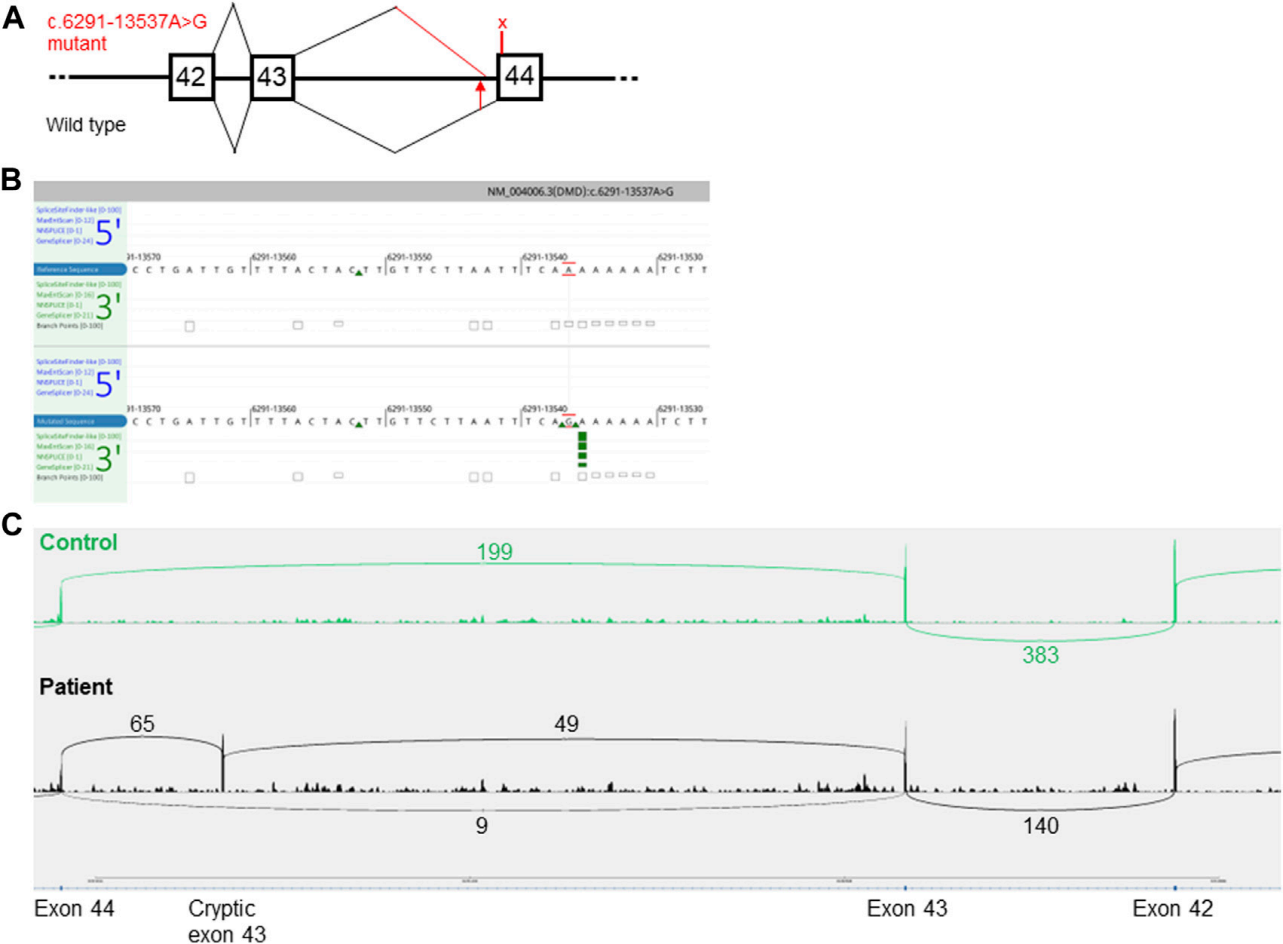
Predicted aberrant splicing in *DMD*. **(A)**, a schematic representation of the wild type (lower part) and the aberrant splicing pattern (upper part) caused by the c.6291–13537A>G variant in *DMD*. The squares with numbers inside denote the coding exon numbers. The position of the variant is marked with a red arrow. The predicted premature stop codon located in the cryptic pseudo-exon in intron 43 p.(Arg2098Lysfs*8), is marked with an X. The figure is not drawn to scale. **(B)**, In silico prediction of a cryptic acceptor splice site (green arrows) in intron 43 of *DMD* caused by the NM_004006.3(*DMD*):c.6291–13537A>G variant (lower panel). Image adapted from Alamut Visual Plus v.1.5.1 (SOPHiA GENETICS) software. **(C)**, Sashimi plot of RNA-sequencing data presenting the read count of wild type *versus* aberrant splice isoforms. Upper part, control muscle biopsy with a normal splicing pattern. The lower part is a muscle biopsy from the patient.

Previous results indicated a possible dystrophin deficiency at the transcript level. Therefore, a biopsy sample from the same muscle as used in 2010 was used to perform mRNA and Western blot analyses (LUMC, Leiden, Netherlands). On Western blot aberrant dystrophin protein expression was observed (Figure 2). No dystrophin expression was detected when using the DYS2 antibody* (reacts strongly with the carboxy terminus, between exon 77 and exon 79), while strongly reduced full-length (400 kilodaltons) dystrophin expression was detected using the DYS1 antibody** (reacts strongly with the rod domain, between exon 26 and exon 30). Isolation of RNA from muscle tissue using the NucleoSpin^®^ RNA XS kit for RNA isolation from small samples (Macherey-Nagel) followed by reverse transcription PCR (rtPCR, details available upon request) of the *DMD* gene and RNA-sequencing using the Takara SMARTer Stranded Total RNA-Seq Kit v3—Pico Input Mammalian Library Prep Kit to process the samples was performed. Sanger sequencing of the rtPCR product and RNA-sequencing using the Illumina NovaSeq6000 (performed at GenomeScan B.V., Netherlands) showed aberrant splicing of the *DMD* gene, where 154 nucleotides from intron 43 were included between exon 43 and 44 resulting in a frameshift and a premature stop codon (Figure 3). Besides the aberrant splicing pattern, also normal splicing of the *DMD* gene was observed (Figures 4A–C). Sequence analysis of genomic DNA extracted from a blood sample revealed that the patient is hemizygous for a cryptic splice variant in intron 43 of the *DMD* gene; (NM_004006.3): c.6291–13537A>G, p.(Arg2098Lysfs*8). The variant is predicted to create a cryptic splice acceptor site in intron 43 resulting in a partial intron retention of 154 nucleotides in agreement with the RNA result (Figure 4C). The c.6291–13537A>G variant in *DMD* is classified as likely pathogenic according to ACMG guidelines (Richards et al., 2015). The clinical diagnosis of BMD was finally genetically confirmed.

### Therapeutic intervention

Unfortunately, there is no gene therapy targeting intron variants as these variants are extremely rare.

## Discussion

This study highlights the challenges encountered in achieving an accurate genetic diagnosis for individuals with suspected Becker muscular dystrophy, particularly when dealing with deep intronic splice-altering variants (Yang et al., 2019; Zimowski et al., 2021; Viggiano et al., 2023).

The patient and his family underwent a prolonged period of waiting for genetic confirmation, during which extensive biochemical, immunohistochemistry and genetic diagnostic testing were conducted. Ultimately, a molecular diagnosis was achieved through sequencing of mRNA extracted from muscle tissue followed by the investigation of a specific intronic area of genomic DNA. This illuminates the importance of available genetic testing facilities that can accommodate RNA analyses as a service or in collaboration with clinical genetic laboratories.

Here, we argue that the availability of resources to link the clinical diagnosis with protein, RNA, and DNA alterations is crucial to confirm the diagnosis (Xie et al., 2021; Segarra-Casas et al., 2023)**.** A genetically verified DMD/BMD diagnosis is important for patient care and, depending on the mutation type, could potentially prove to be eligible for targeted exon-skipping therapies (Sheikh and Yokota, 2020).

Since deletion and duplication of exons are the most common cause of BMD, the recommended diagnostic approach involves initial screening for the relative number of copies of all exons in the gene using quantitative methods, such as multiplex litigation-dependent probe amplification (MLPA) (Birnkrant et al., 2018; Fratter et al., 2020). If no copy number variant is found, genomic sequencing of the *DMD* gene is an appropriate second step (Birnkrant et al., 2018; Fratter et al., 2020). In cases where a strong suspicion of DMD/BMD persists despite negative results, a muscle biopsy is recommended to confirm dystrophinopathy using immuno-histochemistry and Western blot (Birnkrant et al., 2018; Fratter et al., 2020). RNA analysis may reveal unusual variants, such as deep intronic splice-altering variants and complex rearrangements. In summary, more than 99% of genetic diagnoses can be confirmed by using these steps (Xie et al., 2021). Today, we must consider utilizing whole genome sequencing (WGS) for *DMD* diagnostics since WGS can be validated to detect copy number variation even at a single exon resolution.

It is important to confirm the diagnosis using genetic tests to be able to perform carrier- and prenatal testing, offer genetic counseling to the affected individual and his relatives, monitor carriers for cardiomyopathy risk, and offer possible genetic treatments (Zampatti et al., 2018; Sheikh and Yokota, 2020). Frameshift variants in the coding regions of the *DMD* gene are rare in BMD. In this patient, the variant is located deep-intronic. As both normal and abnormal splicing of RNA is observed in this case, the finding is not a typical frameshift but rather a cryptic splice variant.

Deep intronic variants, located outside the 50 nucleotides near the splice sites, are estimated to contribute to up to 7% of BMD/DMD cases, leaving a substantial proportion of dystrophin-deficient patients without a molecular diagnosis following routine genetic testing (Waldrop et al., 2022). Aberrant RNA-transcript can be caused by intronic single nucleotide variants, structural rearrangements, and repeat expansions. A flow chart for genetic testing was recently suggested for MLPA/exome-negative patients. If RNA sequencing indicates pseudoexon or exon skipping, the next step should be WGS. If clipped or non-reference sequences are found, WGS and long-read sequencing are recommended. If an intronic termination is found, WGS and 3′RACE are recommended (Okubo et al., 2023). WGS-based diagnostic tests today can be validated to accurately detect single or multi-exon deletions and duplications as well as variants in or near canonical splice sites. Furthermore, WGS contains the genetic information on intronic variants producing aberrant mRNA splicing. Therefore, we argue that WGS, if available, is the ideal starting point for genetic diagnostic analysis of samples from patients with suspected BMD/DMD to promote patient-focused precision medicine.

Approximately 60 deep intronic variants, outside 50 nucleotides from the splice site, are listed in the HGMD database (accession date 29 July 2023). These are scattered in most of the 76 DMD introns, in which variants are published. The variants have clinically often been associated with DMD, BMD, and intermediate forms. A few cases with limb-girdle phenotype, dilated cardiomyopathy, and hyper-creatine-kinaseemia are also found. Two deep splice acceptor variants in intron 43 have previously been published (Cummings et al., 2017; Cohen et al., 2022; Waldrop et al., 2022), but no deep splice donor mutations. In the previous cases, a total of four individuals, all had a clinical diagnosis of BMD. The mechanism for dystrophinopathy in these four cases seems to be the creation of a pseudo-exon, as in our case. An 11% residual normally spliced dystrophin was found in the only case where this was reported (Waldrop et al., 2022). In our case, a residual of ∼7% of wild-type splicing was found. Hence, it seems that only a small amount of normal dystrophin is required for a BMD phenotype. The rate of normally spliced dystrophin in the diagnosis of DMD/BMD patients with intronic variants ranges from 4% to 27%, while DMD patients showed no detectable dystrophin (Okubo et al., 2023). In the studies of patients with a deep intronic variant in exon 43 no information on results of immunohistochemistry or Western blot was available.

In conclusion, after a long and interrupted investigation, a novel variant of BMD was finally identified using RNA analysis, revealing a deep intronic splice-altering variant.

### Patient perspective

There is currently no cure available for this condition, but many preventive measures are available to maintain physical functions. The patient experienced the emotional burden of living with an undiagnosed genetic disorder for more than a decade, and his family also endured a prolonged period of uncertainty before receiving genetic counseling. The precise diagnosis and the possibility of genetic counseling were a relief for the patient and his family.

During genetic counseling, the patient was informed about the inheritance pattern of the pathogenic variant, indicating that his potential future daughters will be carriers without symptoms or with mild expressions of the disease, while his sons will not inherit the DMD variant and are therefore not at risk for BMD.

Screening for the c.6291–13537A>G variant in *DMD* in DNA from the patient’s healthy mother showed positive carrier status. Hence, she could be offered cardiac surveillance due to the increased risk of cardiomyopathy. Her sister was confirmed negative for the variant. The deceased maternal grandmother did not exhibit clinical signs of dilated cardiomyopathy during her lifetime and was not screened for the variant.

## Data Availability

The original contributions presented in the study are included in the article/Supplementary Materials, further inquiries can be directed to the corresponding author.

## Footnotes

1
http://www.Socialstyrelsen.se/kunskapsstod-och-regler/omraden/sallsynta-halsotillstand/beckers-muskeldystrofi/

## Supplementary material

SUPPLEMENTARY TABLE S1 Historical and current information from different episodes of care organized as a timeline.
