# Supplementary material for: Case report: a novel deep intronic splice-altering variant in DMD as a cause of Becker muscular dystrophy
Source: Front Genet. 2023 Sep 19;14:1226766. doi: 10.3389/fgene.2023.1226766 (PMC10546389; doi:10.3389/fgene.2023.1226766)
Supplement: Supplementary file 1 [file Table1.docx]

| **2003** | **2009–2010** | **2015-2018** | **2021-2022** |
| --- | --- | --- | --- |
| *Age 6*  The onset of walking difficulties | *Age 12-13*  Suspected BMD: Positive Gowers’s sign, pseudo-hypertrophy in the calves lordosis in the lumbar spine. | *Age 18-21*  Still ambulatory but had significant walking difficulties, working part-time repairing vehicles with adapted tasks, and getting a driving license. | *Age 24-25*  Still actively working. The disease has a slow course. Cardiology investigation revealed no gross pathology. A new diagnostic investigation was initiated. |
|  | *Muscle biopsy:*  *M*yopathy with partial dystrophin deficiency. *Western blot:*  Decrease in dystrophin  Multiplex ligation-dependent probe amplification detected no deletions nor duplications in the *DMD* gene. Subsequent sequencing of *DMD* showed no pathogenic variants. | *Western blot:*  Absence of dystrophin 1 and 2, the decreased levels of α-dystroglycan and β-sarcoglycan were considered secondary to dystrophin deficiency. | *Western blot + mRNA-analysis*  Performed on the same muscle biopsy sample as in 2010. Aberrant Dystrophin protein expression was observed (Figure 1). Isolation of RNA from muscle tissue followed by reverse transcription PCR (rtPCR) of the DMD gene. Sanger sequencing of the rtPCR product showed aberrant splicing of the DMD gene, where 154 nucleotides from intron 43 were included between exon 43 and 44 resulting in a frameshift and a premature stop codon (Figure 2). Sequencing analysis of genomic DNA extracted from a blood sample *revealed that the patient is* hemizygous for a cryptic splice variant in intron 43 of the DMD gene. The variant is predicted to create a cryptic splice acceptor site in intron 43 resulting in a partial intron retention of 154 nucleotides in agreement with the RNA result (Figure 3B). |
|  | The clinical diagnosis of BMD could not be genetically confirmed |  | The c.6291-13537A>G variant in DMD is classified as likely pathogenic according to ACMG guidelines (11). The clinical diagnosis of BMD was finally genetically confirmed. (NM_004006.3): c.6291-13537A>G p.(Arg2098Lysfs*8). |
